# Avian Macrophage Responses to Virulent and Avirulent *Clostridium perfringens*

**DOI:** 10.3390/pathogens11010100

**Published:** 2022-01-15

**Authors:** Raveendra R. Kulkarni, Carissa Gaghan, Javid Mohammed

**Affiliations:** Department of Population Health and Pathobiology, College of Veterinary Medicine, North Carolina State University, 1060 William Moore Dr., Raleigh, NC 27606, USA; cegaghan@ncsu.edu (C.G.); jpmohamm@ncsu.edu (J.M.)

**Keywords:** macrophages, *Clostridium perfringens*, virulence, chicken, necrotic enteritis, immune response

## Abstract

The present study evaluated the avian macrophage responses against *Clostridium perfringens* that varied in their ability to cause necrotic enteritis in chickens. Strains CP5 (avirulent-*netB*+), CP1 (virulent-*netB*+), and CP26 (highly virulent-*netB*+*tpeL*+) were used to evaluate their effect on macrophages (MQ-NCSU cells) and primary splenic and cecal tonsil mononuclear cells. The bacilli (whole cells) or their secretory products from all three strains induced a significant increase in the macrophage transcription of Toll-like receptor (TLR)21, TLR2, interleukin (IL)-1β, inducible nitric oxide synthase (iNOS), and CD80 genes as well as their nitric oxide (NO) production and major histocompatibility complex (MHC)-II surface expression compared to an unstimulated control. The CP1 and CP26-induced expression of interferon (IFN)γ, IL-6, CD40 genes, MHC-II upregulation, and NO production was significantly higher than that of CP5 and control groups. Furthermore, splenocytes and cecal tonsillocytes stimulated with bacilli or secretory products from all the strains showed a significant increase in the frequency of macrophages, their surface expression of MHC-II and NO production, while CP26-induced responses were significantly higher for the rest of the groups. In summary, macrophage interaction with *C. perfringens* can lead to cellular activation and, the ability of this pathogen to induce macrophage responses may depend on its level of virulence.

## 1. Introduction

Innate immune defense forms an integral component of host immunity against infectious agents. While several constituents of this component such as cells, receptors, and soluble factors play different roles, macrophages provide a vital first line of defense against pathogens [1]. Macrophages sense microbes through pattern recognition receptors, particularly the Toll-like receptors (TLR), to initiate their functional roles of phagocytosis, antigen presentation, and secretion of cytokines required in initiating an adaptive immune response. The importance of macrophages in the defense against several bacterial pathogens [2,3], including those affecting chickens such as avian pathogenic *Escherichia coli* [4], have been reported. Macrophage responses to *Clostridium perfringens* strains of human origin that cause gas gangrene or food poisoning have also been characterized [5,6]. 

Virulent strains of *Clostridium perfringens* cause necrotic enteritis (NE) in chickens, an economically important disease affecting poultry worldwide. The global annual losses due to NE in 2015 were estimated to be around USD 6 billion [7]. *C. perfringens* uses many virulence strategies that are not only limited to the tissue-degrading toxins such as NetB and TpeL, and possibly alpha-toxin, but also include numerous metabolic enzymes, minor toxins, and adhesion molecules [8]. We have previously shown that antibodies to alpha-toxin and certain metabolic enzymes and proteins are important in NE immunity and hence, these proteins may have a role in NE pathogenesis [9,10]. The pathogenesis of NE is complex, and while NetB has been shown to be a critical virulence factor, TpeL toxin has been reported to enhance the virulence of some *netB+ C. perfringens* strains [11,12]. Recent reports also suggest that there are NE-causing unique strains that possess certain signature NE-associated virulence gene(s) that are absent in commensal avirulent non-NE strains of *C. perfringens* [8,13]. Although the NE pathogenesis is moderately well studied, the basis of immunity and immune responses against *C. perfringens* is poorly understood. 

Chicken macrophages that are professional phagocytes and antigen-presenting cells (APC) are an important cell type in the host immune defense [14]. Although there is no direct evidence of a functional interaction of avian APC with *C. perfringens*, studies that have investigated intestinal immune responses against this pathogen have found increased jejunal or ileal transcription of interleukin (IL)-1β, IL-6, IL-8, and IL-12 genes as well as major histocompatibility complex (MHC) class II gene expression and nitric oxide (NO) production [15,16,17]. Chicken embryonic fibroblasts and HD11 macrophages stimulated with *C. perfringens* have been shown to produce NO in a TLR2- or TLR4-dependent manner [18,19]. However, the *C. perfringens* strain (ATCC 13124) used in that study was not of avian origin. 

In the present study, we used MQ-NCSU chicken macrophage cells as well as primary mononuclear cells isolated from chicken spleen and cecal tonsils and three *netB*+ *C. perfringens* field strains, with or without *tpeL,* that varied in their ability to cause NE in chickens to investigate the following. (1) The interaction of *C. perfringens* bacterial whole cells (termed as ‘bacilli’ hereafter) and their secretory products with MQ-NCSU cells leading to immune gene expression, macrophage activation, and NO production. (2) The interaction of *C. perfringens* bacilli and their secretory products in primary splenocytes and cecal tonsillocytes leading to an augmented macrophage frequency and cellular activation. (3) The effect of the in vivo virulence nature of *C. perfringens* strains on the macrophage responses during in vitro and ex vivo interactions.

## 2. Results

### 2.1. Macrophage Responses to Clostridium Perfringens Whole Cells 

**Figure 1:**
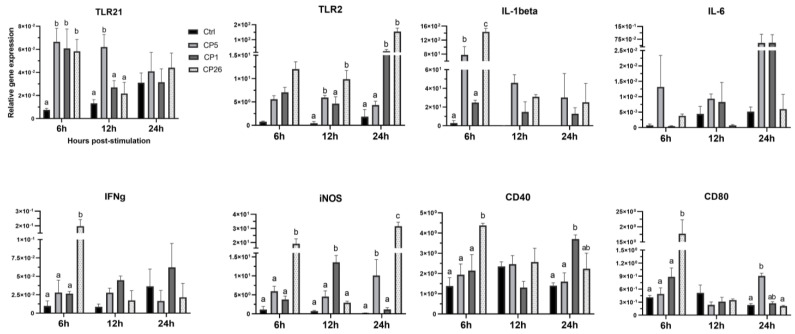
Interaction of macrophages with *C. perfringens* bacilli induces cellular expression of immune genes. MQ−NCSU chicken macrophages were interacted with bacilli (whole cells) of *C. perfringens* strains that varied in their ability to cause necrotic enteritis in chickens, namely, CP5 (*netB*+ avirulent), CP1 (*netB*+ virulent), and CP26 (*netB*+*tpeL*+ highly virulent) for 6, 12 and 24 h. Cells, post-stimulation, were collected in Trizol for RNA extraction and cDNA synthesis. Real-time PCR to quantify the expression of TLR21, TLR2, IL−1β, IL−6, IFNγ, iNOS, CD40, and CD80 genes was performed along with the housekeeping gene (β−actin). The expression levels of the target genes are shown as relative to β−actin. Error bars represent mean value ± standard error. Different letters above the standard error of mean bars within each of the data set graphs indicate significant statistical difference (*p* ≤ 0.05) between the groups.

To evaluate *C. perfringens* bacilli-induced responses in macrophages, the expression of TLR21, TLR2, IL-1β, IL-6, interferon (IFN)γ, inducible nitric oxide synthase (iNOS), CD40, and CD80 genes was quantified at 6, 12, and 24 h post-stimulation. As shown in Figure 1, there was an upregulation (*p* < 0.05) of TLR21 gene expression in all the *C. perfringens* strains-treated groups at 6 h post-stimulation, while CP5 treatment showed a sustained elevation (*p* < 0.05) of TLR21 expression in these cells up to 12 h when compared to unstimulated controls. The transcriptional upregulation of TLR2 was found to be significant (*p* < 0.05) in CP26-treated macrophages at both 12 and 24 h, whereas the expression of this gene in macrophages stimulated with CP5 and CP1 strains was increased (*p* < 0.05) at 12 and 24 h post-stimulation, respectively, only when compared to controls. Treatment of macrophages with strain CP26 also induced an increased (*p* < 0.05) expression of IL-1β, IFNγ, iNOS, CD40, and CD80 genes at 6 h post-stimulation, compared to controls; additionally, the expression of IL-1β at 6 h and iNOS at 24 h was also higher (*p* < 0.05) in comparison to CP5-treated cells. Furthermore, while CP1 treatment induced upregulation (*p* < 0.05) of iNOS at 12 h (compared to the control) and CD40 at 24 h (compared to CP5 and the control), the CP5 strain induced increased (*p* < 0.05) iNOS (compared to CP1 and control) and CD80 (compared to control and CP26) transcription at 24 h post-stimulation.

### 2.2. Macrophage Responses to Clostridium Perfringens Secretory Products

**Figure 2:**
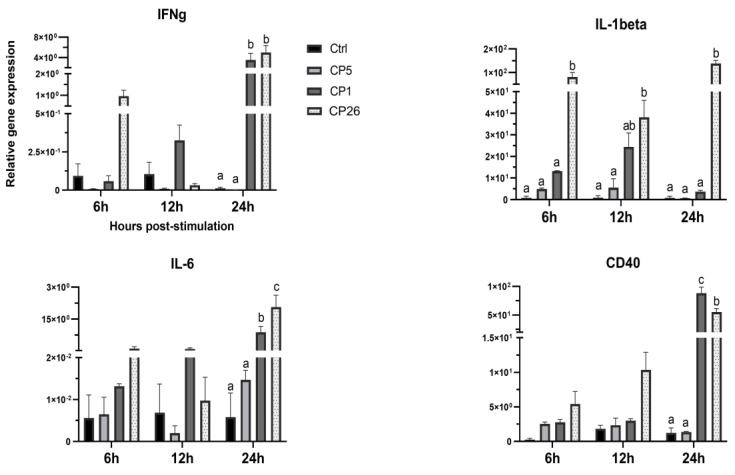
Interaction of macrophages with *C. perfringens* secretory products induces cellular expression of immune genes. MQ−NCSU chicken macrophages were interacted with secretory products from *C. perfringens* strains that varied in their ability to cause necrotic enteritis in chickens, namely, CP5 (*netB*+ avirulent), CP1 (*netB*+ virulent), and CP26 (*netB*+*tpeL*+ highly virulent) for 6, 12, and 24 h. Cells, post-stimulation, were collected in TRIzol for RNA extraction and cDNA synthesis. Real-time PCR to quantify the expression of IL−1β, IL−6, IFNγ, and CD40 was performed along with the housekeeping gene (β−actin). The expression levels of the target genes are shown as relative to β−actin. Error bars represent mean value ± standard error. Different letters above the standard error of mean bars within each of the data set graphs indicate significant statistical difference (*p* ≤ 0.05) between the groups.

The stimulatory effects of *C. perfringens* secretory products on macrophages were evaluated based on the cellular expression of immune-related genes that included IL-1β, IL-6, IFNγ, TLR21, TLR2, iNOS, CD40, and CD80 at 6, 12, and 24 h post-stimulation. Three supernatant dilutions (1:2, 1:5, and 1:10) were used to stimulate macrophages, of which, 1:5 was found to be optimal based on the cell death and stimulatory potential. Figure 2 depicts the experiments that used 1:5 dilution of the *C. perfringens* secretory components. Macrophages treated with CP1 and CP26 secretory products had a higher (*p* < 0.05) expression of IL-6, IFNγ, and CD40 genes compared to CP5 and unstimulated control groups at 24 h post-stimulation (Figure 2). While strain CP26-induced IL-6 gene expression in macrophages was also higher (*p* < 0.05) than that of CP1-treated cells, the CD40 transcriptional upregulation in CP1-treated cells was higher (*p* < 0.05) compared to the CP26-treated group at 24 h post-stimulation. Additionally, the CP26 secretory products-induced transcription of IL-1β was higher (*p* < 0.05) than CP5 and controls at 6, 12, and 24 h and that of CP1 at 6 and 24 h post-stimulation (Figure 2). No significant differences in the expression of other above-mentioned genes were observed (data not shown).

**Figure 3:**
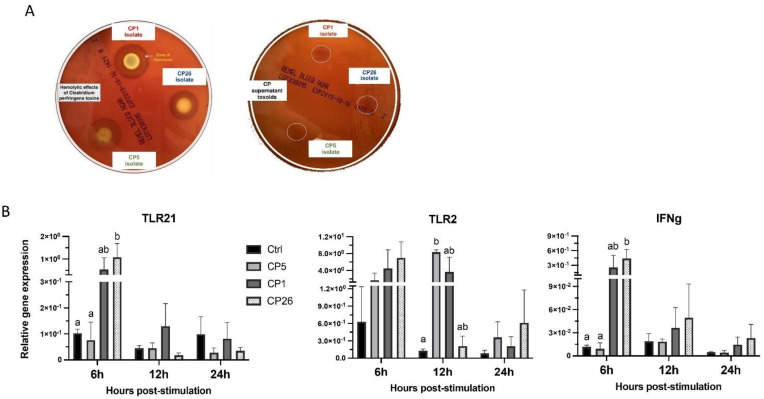
Interaction of macrophages with *C. perfringens* supernatant toxoid induces cellular expression of immune genes. MQ−NCSU chicken macrophages were interacted with toxoided secretory products (Panel **A**) from *C. perfringens* strains that varied in their ability to cause necrotic enteritis in chickens, namely, CP5 (*netB*+ avirulent), CP1 (*netB*+ virulent), and CP26 (*netB*+*tpeL*+ highly virulent) for 6, 12, and 24 h. In (Panel **B**), cells, were collected following to stimulation in Trizol for RNA extraction and cDNA synthesis. Real-time PCR to quantify the expression of TLR21, TLR2, and IFNγ was performed along with the housekeeping gene (β−actin). The expression levels of the target genes are shown as relative to β−actin. Error bars represent mean value ± standard error. Different letters above the standard error of mean bars within each of the data set graphs indicate significant statistical difference (*p* ≤ 0.05) between the groups.

To determine if the neutralization activity in the supernatant component could induce a different cellular response phenotype, the macrophages were treated with supernatant toxoids (Figure 3a). The immune gene expression analysis showed that CP26 secretory toxoid could induce an upregulation (*p* < 0.05) of TLR21 and IFNγ transcription compared to CP5 and control-treated cells at 6 h post-stimulation (Figure 3b). Furthermore, the CP5 secretory toxoid-treated macrophages were found to have higher (*p* < 0.05) expression of TLR2 gene at 12 h post-stimulation when compared to unstimulated controls (Figure 3B). No significant differences in the expression of other immune-related genes were observed (data not shown).

### 2.3. Macrophage Expression of MHC-II and Production of Nitric Oxide

**Figure 4:**
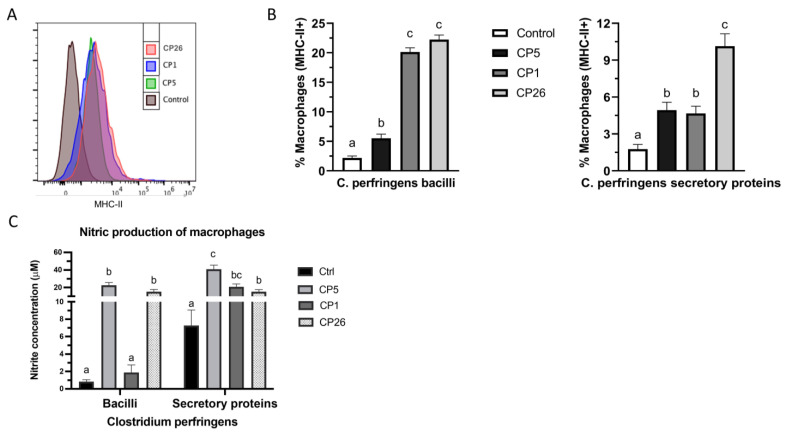
Interaction of macrophages with *C. perfringens* bacilli or secretory products induces cellular upregulation of MHC−II expression and nitric oxide production. MQ−NCSU chicken macrophages were stimulated with bacilli or secretory products from *C. perfringens* strains that varied in their ability to cause necrotic enteritis in chickens, namely, CP5 (*netB*+ avirulent), CP1 (*netB*+ virulent), and CP26 (*netB*+*tpeL*+ highly virulent) for 24 h. Cells, post-stimulation, were collected for MHC−II surface expression by flow cytometry analysis and the cell culture supernatants were collected for determining NO production by Griess assay. (Panel **A**) depicts the representative histogram plots showing MHC−II expression on macrophages stimulated with *C. perfringens* strains. (Panel **B**) represents the frequency of macrophages upregulating MHC−II in different treatment groups. (Panel **C**) shows NO production by macrophages in response to interaction with *C. perfringens* bacilli or secretory products. Error bars represent mean value ± standard error. Different letters above the standard error of mean bars within each of the data set graphs indicate significant statistical difference (*p* ≤ 0.05) between the groups.

In vitro induction of macrophage activation by *C. perfringens* bacilli or their secretory products was evaluated as measured by their surface upregulation of MHC-II antigen presenting molecules as well as their production of NO. As depicted in Figure 4B, all *C. perfringens* strains, both whole cell and their secretory products, induced an upregulation (*p* < 0.05) in the surface expression of MHC-II molecule when compared to the unstimulated control. Furthermore, while the CP26 and CP1 bacilli-induced MHC-II expression was higher (*p* < 0.05) than CP5, the CP26 secretory products-induced MHC-II upregulation was higher (*p* < 0.05) when compared to that of CP5 and CP1 secretory products.

Analysis of the NO production by macrophages showed that CP5 and CP26 bacilli treatment of cells induced higher (*p* < 0.05) amounts of NO compared to CP1 and control-treated macrophages (Figure 4c). Furthermore, while the secretory components of all three strains showed higher (*p* < 0.05) induction of NO production compared to unstimulated cells, the macrophages treated with CP5-derived secretory products produced higher (*p* < 0.05) levels of NO when compared to the CP26 group. 

### 2.4. Ex Vivo Cellular Responses and Nitric Oxide Production

**Figure 5:**
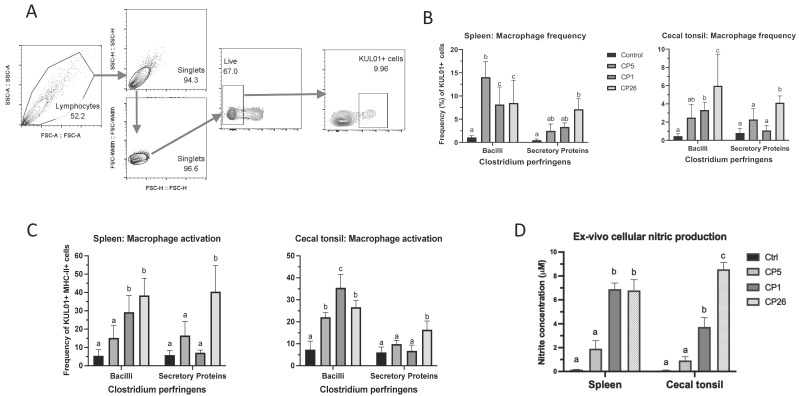
Interaction of splenocytes and cecal tonsillocytes with *C. perfringens* bacilli or secretory products leads to enhanced macrophage response and nitric oxide production. Spleens and cecal tonsils from 3-week-old healthy broiler chickens were collected and mononuclear cell suspensions were prepared. Cells were stimulated with bacilli or secretory products from *C. perfringens* strains that varied in their ability to cause necrotic enteritis in chickens, namely, CP5 (*netB*+ avirulent), CP1 (*netB*+ virulent), and CP26 (*netB*+*tpeL*+ highly virulent) for 24 h followed by staining with antibodies against chicken KUL01 (monocyte/macrophage lineage marker) and MHC−II molecules for flow cytometry analysis. Gating strategy is given in (panel **A**). The total macrophage frequencies and those expressing MHC-II are shown in (panels **B**,**C**), respectively. (Panel **D**) shows NO production by macrophages in response to interaction with *C. perfringens* bacilli. Results from one of the two independent experiments are shown here. Error bars represent mean value ± standard error. Different letters above the standard error of mean bars within each of the cell-type populations indicate the differences were statistically significant (*p* ≤ 0.05).

To evaluate primary macrophage cellular responses to *C. perfringens* and their secretory products, the spleens and cecal tonsils from broiler chickens were harvested and processed to obtain mononuclear single-cell suspensions of splenocytes and cecal tonsillocytes and used in the cell culture experiments. The gating strategy used in immunophenotyping analysis is shown in Figure 5a. The responses were measured by the enumeration of total macrophages (KUL01+ cells) and activated macrophages (KUL01+MHC-II+ cells) as frequencies within the mononuclear cell populations of spleen and cecal tonsils in response to *C. perfringens* stimulation. As depicted in Figure 5b, all *C. perfringens* strains (whole cells) induced an increase (*p* < 0.05) in the splenic macrophage frequency when compared to an unstimulated control. However, these numbers in CP5-treated groups were higher (*p* < 0.05) than the CP1- and CP26-treated cells. The stimulation of cecal tonsillocytes with CP1 and CP26 cells also led to an increase (*p* < 0.05) in the macrophage frequency when compared to an unstimulated control. The macrophage numbers in CP26-treated groups were also higher (*p* < 0.05) than the CP5- and CP1-treated cells. Furthermore, an increase in the splenic macrophage frequency in the group receiving CP26 secretory products was higher (*p* < 0.05) than the control, whereas the cecal tonsil macrophage frequency in this group was also higher (*p* < 0.05) when compared to the control, CP5, and CP1 groups (Figure 5B). 

Analysis of the surface MHC-II expression of macrophages showed that while CP1- and CP26-treated groups had a higher (*p* < 0.05) frequency of activated splenic macrophages (KUL01+MHC-II+), all strains induced an increased number of activated macrophages within the cecal tonsillocyte population when compared to an unstimulated control group (Figure 5c). Additionally, these numbers in the CP1-treated cecal tonsillocytes were also higher (*p* < 0.05) than the CP5 and CP26 bacilli-treated groups. Furthermore, the group receiving CP26 secretory products had a higher (*p* < 0.05) frequency of both splenic- and cecal tonsil-activated macrophages compared to the rest of the groups (Figure 5c).

Additionally, NO production in the culture supernatants was quantified as an indirect measure of macrophage activation. CP1 and CP26 bacilli stimulation of splenic and cecal tonsil cells led to higher (*p* < 0.05) amounts of NO production compared to CP5 and control groups (Figure 5c). The NO production by cecal tonsillocytes receiving CP26 bacilli was also higher (*p* < 0.05) when compared to the CP1 group (Figure 5c). The NO production by either splenic or cecal tonsil cells stimulated with *C. perfringens* secretory products was very low and no significant difference was observed between the treatment groups (data not shown).

## 3. Discussion

Macrophages play a vital role in innate immune defense against a variety of pathogens affecting humans and animals, including poultry [1]. The present study evaluated macrophage responses against virulent and avirulent chicken strains of *C. perfringens*. The findings showed that the interaction of macrophages with *C. perfringens* can induce cellular activation, and the ability of this pathogen to activate macrophages depended on the level of virulence with the two NE-producing strains used in the present study inducing a more robust macrophage activation than the avirulent strain.

Professional phagocytes such as macrophages and dendritic cells that can recognize and phagocytose pathogens and present microbial antigens to T cells play a key role in the early combat against pathogens and in initiating adaptive responses [14]. Information related to macrophage interaction with certain avian pathogens is available [20,21]; however, *C. perfringens* interaction with these cells is poorly understood. The present study’s findings showed that bacilli from all three strains induced a significant increase in the transcription of TLR21 and TLR2 receptors, IL-6 and IL-1β cytokines, and CD80 costimulatory genes in macrophages compared to an unstimulated control. It can be suggested that avian macrophages can use TLR2 and TLR21 microbe sensing receptors to recognize *C. perfringens* to exhibit a proinflammatory response. Previous studies investigating the intestinal gene expression profile in response to *C. perfringens* infection in chickens have also generally shown increased transcription of TLR2, IL-1β, IL-6, and IL-8 [15,22], while the recognition of a commensal clostridial species, *C. butyricum*, by human epithelial cells has been suggested to occur via TLR2 [23]. Similarly, bone marrow-derived chicken macrophages or HD-11 cells stimulated with avian enteropathogenic *E. coli* (APEC) and bacterial lipopolysaccharide, respectively, have also been shown to upregulate IL-8, IL-12, IL-6, and IL-1β mRNA expression [21,24]. Interestingly, the expression of IFNγ cytokine and CD40 costimulatory genes induced by the *netB*^+^ virulent strains, particularly those carrying additional *tpeL* toxin gene (CP26), was significantly higher than avirulent CP5 and controls. These findings imply that macrophages seem to respond to virulent strains in a more robust manner compared to avirulent strains since IFNγ and CD40 expression are important indicators of enhanced cellular activation, pathogen killing, and T cell co-stimulation [3]. Furthermore, our observation that macrophage transcription of iNOS was induced as early as 6 h post-stimulation by all the strains was further supported by their NO production. This observation is in agreement with previous studies that showed interaction of chicken embryo fibroblasts with *C. perfringens* or APEC stimulation of primary macrophages can lead to cellular activation and NO production [20,21]. However, the finding from the present study that virulent strains can induce a robust macrophage activation compared to an avirulent strain suggests that virulence of the pathogen, determined by its ability to cause NE experimentally, seems to positively influence macrophage activation and their immune function. This response by macrophages likely relates to the functional expression of NetB toxin in the two virulent strains used in the present study, which needs further investigative confirmation.

One of the important *C. perfringens* factors known to be vital to NE pathogenesis and immunity is the pathogen-derived secretory products [8]. The pathogenesis of, and immunity to NE is not only limited to *C. perfringens*-secreted tissue-degrading toxins such as netB, TpeL and, perhaps, alpha-toxin but also certain metalloproteases, minor toxins, adhesins, and numerous metabolic and degradative enzymes [10]. The present study that employed *C. perfringens* secretory component to stimulate macrophages found that while only virulent *C. perfringens* strain-derived secretory products could induce significantly higher expression of IFNγ, IL-6, and CD40 genes, the highly virulent strain (CP26) alone could induce a significantly elevated level of IL1β transcripts. Furthermore, the MHC-II surface expression of macrophages by CP26-secreted products was significantly higher than the virulent CP1 and avirulent CP5 groups. This observation suggests that secreted products from virulent *C. perfringens*, particularly the highly virulent strain, may contain factors that are immunostimulatory and possess an ability to activate macrophages in facilitating an innate inflammatory response against the pathogen. A study by Guo et al. (2015) demonstrated the ability of avian *C. perfringens* alpha-toxin in inducing an inflammatory response characterized by elevated levels of IL-6, IL-8, and iNOS transcription [16]. Vale et al. (2018) also suggested that bacterial toxins and other virulence proteins can enzymatically modify cellular targets to induce immune cell signaling, leading to an inflammatory response [25]. Additionally, when the supernatant toxoids were used in the present study, the highly virulent CP26 strain induced a significantly upregulated TLR21 and IFNγ transcription compared to avirulent and control groups. Based on this observation, it was evident that toxoiding had no effect on the ability of CP26 to enhance IFNγ transcription, while TLR21 expression in macrophages required the neutralization of toxin activity in the secreted component. Previous reports have shown that secreted factors such as alpha-toxin from *C. perfringens* (gas gangrene strain), binary toxin (CDT) from *Clostridioides difficile*, or type III secretion factors from Gram negative bacteria can trigger TLR-mediated recognition in immune cells to induce an inflammatory response [26,27,28,29]. To this end, further studies are essential to determine which of the secreted factors can specifically target macrophage activation machinery. 

During the in vivo process of infection and immunity, macrophages respond to bacteria or their secretory products by proliferating and upregulating their surface expression of the MHC-II molecule for antigen presentation to T cells [30]. The present study found that the virulent strains, when compared to avirulent strains, could induce a significantly higher frequency of both splenic and cecal tonsil macrophages as well as their upregulation of MHC-II molecules. Furthermore, secreted products from the highly virulent CP26 strain alone showed augmented proliferation of macrophages and their MHC-II expression, suggesting that virulent *C. perfringens* can trigger macrophage responses in both local mucosal as well as systemic lymphoid tissues. A previous study has shown that chicken bone marrow cultures predominantly containing dendritic cells can respond to bacterial stimulation by upregulating the expression of their costimulatory molecules that included MHC-II, CD40, CD80, and CD86 [31]. A recent study that investigated the ex vivo effects of *Campylobacter jejuni* on the chicken splenic and cecal tonsil cells showed a significant proliferation of mononuclear cells in response to *C. jejuni* or concanavalin A mitogen, suggesting a pathogen-induced immunostimulation [32]. Additionally, the authors found that splenocyte expression of the iNOS gene and subsequent production of NO was elevated in response to *C. jejuni* treatment. The present study also found that NO production in the splenocyte and cecal tonsillocyte culture supernatants were significantly higher in the virulent *C. perfringens*-stimulated groups compared to avirulent CP5 and that the ability of CP26 to induce NO production by cecal tonsil cells was superior to CP1 virulent strain. Although *C. perfringens* is an enteric pathogen, we sought to stimulate splenic cells since it is known that compromised intestinal barrier functions during the necrotic events induced by virulent strains can facilitate this pathogen and their secretory factors to reach systemic organs [33,34]. Taken together, our observations suggest that while the virulent *C. perfringens* can trigger activation of both cecal tonsil and splenic macrophages that are the predominant cellular source of NO production [35], the mucosal lymphoid tissues such as cecal tonsils seem to exhibit an even stronger response against highly virulent *C. perfringens* strain [36]. 

An interesting finding that was consistent throughout the present study was that virulent *C. perfringens* bacilli or their secretory products could induce a stronger immune activation of macrophages as well as primary splenocytes and cecal tonsillocytes. This ability to activate chicken immune cells in the highly virulent strain was even stronger. One possible reason could be that the virulent strains, particularly the highly virulent avian *C. perfringens*, such as the human gas gangrene (Str. 13) or food poisoning (SM101) strains, may be equipped with immunoevasive strategies such as spore formation to evade phagolysosomal killing by macrophages so that these cells mount a robust activation and inflammatory responses against this pathogen [5,6,37]. The other possibility is that for the virulent strains, being capable of secreting higher amounts of immunoreactive proteins that include toxins and enzymes, may work to their advantage to resist or delay respiratory burst activity within the macrophages. We have found previously that virulent *C. perfringens* produce higher levels of immunoreactive proteins than avirulent strains [9]. Additionally, a similar suggestion has been made previously by a study that showed virulent (wild-type) strains of duck hepatitis A virus or infectious pancreatic necrosis virus were capable of establishing a successful infection that could trigger enhanced responses in chicken cells compared with the attenuated strains [38,39]. However, further research is required to investigate the mechanistic interaction of macrophages with avian *C. perfringens* strains, including the immunoevasive property used by this pathogen, if any. Lastly, it is noteworthy here that the present study used only two virulent and one avirulent strains to study the possible effects of *C. perfringens* on macrophage responses in vitro and ex vivo. Although the findings presented here provide important clues as to the subtle influence of *C. perfringens’* virulence on immune cell activation, future work employing more strains with proven NE-producing ability is certainly warranted to draw supplementary definitive conclusions.

## 4. Materials and Methods

### 4.1. Cells

MQ-NCSU cells, a chicken macrophage cell line [40], were kindly provided by Dr. Matthew Koci, College of Agriculture and Life Sciences, NC State University, and used to study interaction of macrophages with *C. perfringens*. The cells were maintained and cultured at 40 °C with 5% CO_2_ in LM medium, containing: 1:1 combination of McCoy’s 5A modified medium and L-15 Leibovitz medium supplemented with 8% fetal bovine serum, 10% chicken serum, 1% tryptose phosphate broth, 1% sodium pyruvate, 2 mM L-glutamine, and antimicrobial mixture of penicillin–streptomycin and amphotericin B. In all the experiments, about 5 × 10^5^ cells were seeded per well in a 24-well plate to grow overnight to allow for a confluent monolayer before stimulating with various *C. perfringens* bacilli or their secretory products. The treatment of cells with *C. perfringens* was performed using DMEM containing 10% FBS, and virus infection was carried out using DMEM containing 2.5% BSA, 2.5% HEPES, 1% pen/strep, gentamicin, and 0.04% trypsin/TPCK.

### 4.2. Bacteria

Three *C. perfringens* strains, CP5, CP1, and CP26, isolated from chickens and used in the present study, were kindly provided by Dr. John F Prescott, University of Guelph, Canada. While both CP5 and CP1 (clinical NE isolate) were *netB*+*tpeL*-, CP5 was found to be avirulent and CP1 virulent in our previous chicken challenge experiments [41]. The CP26 strain isolated from chicken at slaughter [42] was *netB*+*tpeL*+ and was found more virulent (referred to as ‘highly virulent’ [11] in this manuscript) than CP1 in our chicken experiments. *C. perfringens* were grown in cooked meat medium (Difco) for 24 h at 37 °C. Fluid thioglycollate medium (Difco) was then inoculated with a 3% (vol/vol) inoculum from the *C. perfringens*-infected cooked meat medium and incubated at 37 °C for 16 h to obtain a growth for CP5, CP1, and CP26 strains that was 5.8–8.9 × 10^8^ colony forming units (CFU).

### 4.3. Secretory Proteins Preparation

For collecting *C. perfringens* supernatant products, a previously described method was followed [9]. Briefly, the bacteria were grown in a fluid thioglycollate medium and the culture supernatants were concentrated (20×) and dialyzed using 10 kDa cutoff Amicon centrifugal filter units (Millipore Inc., Billerica, MA, USA) to obtain secreted products. The macrophages (MQ-NCSU cells), primary splenocytes, or tonsillocytes were stimulated with the concentrated secretory supernatant at 1:2, 1:5, or 1:10 dilution ratios in DMEM (for MQ-NCSU) or RPMI (for primary chicken spleen or cecal tonsil cells) media. Using each of these dilution ratios, the cells were stimulated for 6, 12, or 24 h.

To neutralize the *C. perfringens* toxin activity in the culture supernatants, toxoid preparation was carried out following the method described previously [43] with minor modifications. A total of 10 mg of non-toxoid concentrated secretory protein preparation was reconstituted at 500 μg/mL in 1× PBS and formaldehyde solution was added at 1% of the final volume. After incubation for 5 days at 37 °C, the reaction was stopped by the addition of l-lysine (30 mM final concentration) and the residual formaldehyde was removed by dialysis overnight against PBS by using Amicon centrifugal filter units. The absence of toxin-mediated hemolytic activity was confirmed by spotting the toxoid preparations along with the non-toxoid secretory preparations on blood agar plates (Figure 3a). The macrophages were stimulated for 6, 12, or 24 h with the toxoid preparations at 50, 250, or 500 μg protein/mL in DMEM media.

### 4.4. Animals

Ross 708 male broilers used in the ex vivo immune evaluation experiment were procured from the North Carolina State University Broiler Breeder Flock and housed in the University animal isolation facility for three weeks. The birds were reared in floor pens in wood shavings with unlimited access to food and water. At 21 days of age, 3-week-old healthy birds with no apparent enteric lesions were humanely euthanized to collect spleen and cecal tonsils.

### 4.5. In Vitro Cellular Stimulation

MQ-NCSU macrophages were stimulated with CP5, CP1, and CP26 strains of *C. perfringens* bacilli or their secretory products along with an unstimulated or medium-only control group. For stimulation with *C. perfringens* bacilli, a pilot growth curve study was conducted to obtain CFU corresponding to optical density (OD) of *C. perfringens* culture. A treatment dose-range finding experiment was carried out to determine an optimal CFU needed for macrophage stimulation without considerable cell death. Based on the pilot experiment, 1000 bacteria per cell culture well was chosen for all the cell stimulation experiments described in this study. Cells were thus stimulated with *C. perfringens* bacilli or secretory products (concentrations mentioned above) for 6, 12, and 24 h for immune gene expression analysis, while for flow cytometry and NO production analysis, the stimulation period was 24 h.

### 4.6. Ex Vivo Cellular Stimulation 

To obtain primary chicken cells for ex vivo cell stimulation assay, the spleen and cecal tonsil tissues were collected and single-cell suspensions of splenocytes and cecal tonsillocytes were prepared following a protocol as described previously [44]. Briefly, the tissues were rinsed with 1× HBSS and then pestled directly on a 40 μm cell strainer using the flat end of a disposable 5 mL syringe. Cells were passed through the strainer with RPMI complete medium and centrifuged at 400× *g* for 10 min at 4 °C to pellet the cells. The RBC lysis step was performed using the ACK lysis buffer (Lonza, CA, USA). The cell preparation devoid of erythrocytes was again passed through 40 μm cell strainer to exclude clumps that may interfere during cell stimulation and the purified mononuclear cell preparation was counted using a hemocytometer and Trypan blue, and cell density was adjusted to a final concentration of 1 × 10^7^ cells/mL before setting the cells for stimulation at 1 × 10^6^/well. Cells were thus stimulated with *C. perfringens* bacilli or secretory products, as described above, for a period of 24 h to collect cells for flow cytometry and the cell supernatants for NO production analysis.

### 4.7. Immune Gene Expression

Macrophages were stimulated with *C. perfringens* bacilli or their secretory products for 6, 12, and 24 h and the cells were collected, and total RNA was extracted using TRIzol reagent (Invitrogen, Carlsbad, CA, USA) according to the manufacturer’s protocol before being treated with a DNA-free Kit (Invitrogen, Carlsbad, CA, USA). Subsequently, cDNA synthesis was performed with 500–1000 ng of purified RNA using a High Capacity RNA-to-cDNA kit (Applied Biosystems, Waltham, MA, USA) according to the manufacturer’s recommended protocol. The resulting cDNA was subsequently diluted 1:10 in nuclease free water for real-time PCR procedures.

Quantitative real-time reverse-transcriptase PCR using SYBR Green was performed on diluted cDNA using a QuantStudio 6 Flex System and QuantStudio Real-Time PCR Software (Applied Biosystems, Waltham, MA, USA). Briefly, each reaction involved a pre-incubation period of 50 °C for two minutes followed by 95 °C for two minutes, followed by 40–50 cycles of 95 °C for 10 s, 55–64 °C for 5 s, depending on the primers binding suitability. The elongation step was 72 °C for 10 s. Subsequent melt curve analysis was performed by heating to 95 °C for 15 s, cooling to 60 °C for 1 min, and heating to 95 °C for 15 s. Primers for the amplification of β-actin [44], TLR21 [22], TLR2 [22], IL-1β [22], IL-6 [44], IFNγ [44], iNOS [16], CD40 [31], and CD80 [31] genes were synthesized by Integrated DNA Technologies (Coralville, IA, USA), and the primer sequences are given in Table 1. Relative expression levels of all target genes were calculated relative to the housekeeping gene β-actin using a previously described formula [45].

### 4.8. Flow Cytometry

Cells post-stimulation were collected and single-cell suspensions were prepared for immunophenotyping by flow cytometry. Briefly, cells were plated on 96-well round-bottom plates with each well containing 10^6^ cells in 100 μL FACS buffer (PBS with 1% BSA). Primary antibodies were added to each well (0.5–1 μg/10^6^ cells) and stained for 30 min on ice with fluorescent mouse monoclonal antibodies directed to bind chicken CD45 leukocyte marker (clone LT40), monocyte/macrophage (clone KUL01) and MHC-II-PE (2G11) obtained from Southern Biotech Inc., Birmingham, AL, USA. The Invitrogen Live/Dead fixable near-IR staining was additionally used to exclude dead cells during data acquisition and subsequent analysis. The cells were then washed twice in FACS buffer and fixed in 4% paraformaldehyde (PFA) before immunophenotyping analysis. Flow cytometry was performed using LSR-II flow-cytometer (BD Bioscience, San Jose, CA, USA) and data were analyzed using FlowJo Software (v.10). The gating strategy included removal of doublets using FSC-H/W and SSC H/W scatter plots and excluding dead cells followed by gating on CD45+ cells as the backbone for macrophage analysis (Figure 5A. Data analysis was carried out using the FlowJo software (Tree Starr, Ashland, OR, USA).

### 4.9. Nitric Oxide (NO) Measurement

Cells were stimulated with *C. perfringens* or their secretory products for 24 h and the culture supernatants were collected for nitric oxide quantitation. NO production was measured by Griess assay using a commercial kit, according to the manufacturer’s protocol (Promega, Madison, WI, USA). Briefly, 50 μL of cell culture supernatant was transferred to another 96-well plate and 50 μL of modified Griess reagent added to a total volume of 100 μL. After 15 min of incubation at room temperature, optical density (OD) at 530 nm was measured and the concentration of released NO_2_^−^ was extrapolated from the NaNO_2_ standard curve.

### 4.10. Data Analysis

The gene expression and flow cytometry data were analyzed by two-way ANOVA (Tukey’s multiple comparisons test) using GraphPad Prism V9.2 (GraphPad software, San Diego, CA, USA). Data were presented as mean ± standard error of the mean (SEM) and the level of statistical significance considered was at *p* ≤ 0.05.

## 5. Conclusions

In summary, the present study findings demonstrate that the interaction of avian macrophages with *C. perfringens* can induce cellular responses leading to macrophage activation, as determined by their immune gene expression, cellular frequency, upregulation of MHC-II, and NO production. Additionally, our experimental observations indicate that the ability of *C. perfringens* in inducing macrophage responses is a function of their ability to cause NE, suggesting that immune cell activation may have a role in NE that needs further investigation.

## Figures and Tables

**Table 1:** Primers for genes used in real-time quantitative PCR.

Target Gene	Primer Sequence (5′-3′)	Annealing Temp	GeneBank Accession Number
TLR2	F-5′-ATCCTGCTGGAGCCCATTCAGAG3′ R-5′-TTGCTCTTCATCAGGAGGCCACT-3′	60	NM_204278.1
TLR21	F-5′-CCTGCGCAAGTGTCCGCTCA-3′ R-5′-GCCCCAGGTCCAGGAAGCAG-3′	60	NM_001030558.1
IL-1β	F-5′-GTGAGGCTCAACATTGCGCTGTA-3′ R-5′-TGTCCAGGCGGTAGAAGATGAAG-3′	64	AJ009800
IL-6	F-5′- CGTGTGCGAGAACAGCATGGAGA-3′ R-5′-TCAGGCATTTCTCCTCGTCGAAGC-3′	60	NM_204628.1
IFNγ	F-5′-ACACTGACAAGTCAAAGCCGCACA-3′ R-5′-AGTCGTTCATCGGGAGCTTGGC-3′	60	X99774
iNOS	F-5′-GGCAGCAGCGTCTCTATGACTTG-3′ R-5′-GACTTTAGGCTGCCCAGGTTG-3′	64	NM 204961
CD80	F-5′-CTGTTCCTTCACATCCTGAGAG-3′ R-5′-CTTCAACACCATCTATTTGCCAG-3′	58	NM_001079739
CD40	F-5′-CCTGGTGATGCTGTGAATTG-3′ R-5′-CTTCTGTGTCGTTGCATTCAG-3′	58	NM_204665
β-actin	F-5′-CAACACAGTGCTGTCTGGTGGTA-3′ R-5′-ATCGTACTCCTGCTTGCTGATCC-3′	58	X00182

## Data Availability

Not applicable.
